# Oviduct epithelium induces interferon-tau in bovine Day-4 embryos, which generates an anti-inflammatory response in immune cells

**DOI:** 10.1038/s41598-018-26224-8

**Published:** 2018-05-18

**Authors:** Anup K. Talukder, Mohammad B. Rashid, Mohamed S. Yousef, Kazuya Kusama, Takashi Shimizu, Masayuki Shimada, Susan S. Suarez, Kazuhiko Imakawa, Akio Miyamoto

**Affiliations:** 1 Graduate School of Animal and Food Hygiene, Obihiro University of Agriculture and Veterinary Medicine, Obihiro, 080-8555 Japan; 2 Department of Gynecology, Obstetrics and Reproductive Health, Faculty of Veterinary Medicine and Animal Science, Bangabandhu Sheikh Mujibur Rahman Agricultural University, Gazipur, 1706 Bangladesh; 3 Department of Physiology and Pharmacology, Faculty of Veterinary and Animal Science, Hajee Mohammad Danesh Science and Technology University, Dinajpur, 5200 Bangladesh; 4 Department of Theriogenology, Faculty of Veterinary Medicine, Assiut University, Assiut, Egypt; 5 Animal Resource Science Center, Graduate School of Agricultural and Life Sciences, The University of Tokyo, Ibaraki, 319-0206 Japan; 6 Graduate School of Biosphere Science, Hiroshima University, Higashi-Hiroshima, 739-8528 Japan; 7 Department of Biomedical Sciences, Cornell University, Ithaca, NY 14853 USA

## Abstract

Recent studies indicate that communication between the bovine embryo and the mother begins in the oviduct. Here, we aimed to investigate the effect of embryos on bovine oviducts for their immune responses using an *in vitro* model. First, zygotes were cultured with or without bovine oviduct epithelial cells (BOECs) for 4 days, when embryos had reached the 16-cell stage. At that time, we detected interferon-tau (IFNT) in embryos co-cultured with BOECs, but not in embryos cultured alone. Next, peripheral blood mononuclear cells (PBMCs) were incubated either in media from embryo alone cultures or from co-cultures of embryos with BOECs. The medium from embryo alone cultures did not modulate PBMCs gene expression; whereas the embryo-BOEC co-culture medium increased interferon-stimulated genes (*ISGs*: *ISG15*, *OAS1, MX2*), *STAT1*, *PTGES* and *TGFB1* but suppressed *IL17* expression in PBMCs. Both IFNT-treated BOEC culture medium and IFNT-supplemented fresh medium alone without BOEC, modulated PBMCs gene expressions similar to those by the embryo-BOEC co-culture medium. Further, specific antibody to IFNT neutralized the effect of embryo-BOEC co-culture medium on PBMCs gene expression. Our results indicate that BOECs stimulate embryos to produce IFNT, which then acts on immune cells to promote an anti-inflammatory response in the oviduct.

## Introduction

The oviduct is a key organ responsible for final maturation of oocytes, transport of gametes, sperm capacitation, fertilization, and early embryo development^1,2^. The mucosal surface of the oviduct can be exposed to pathogens and endotoxins entering from the uterus, peritoneal cavity, and follicular fluid^3^. Moreover, the oviduct mucosa comes in contact with allogenic sperm and semi-allogenic embryos following insemination in the cow. Thus, the bovine oviduct should be equipped with an efficient and strictly-controlled immune system for allowing sperm transport and early embryonic development, while providing protection against pathogens. Recently, we demonstrated that a pro-inflammatory response (*NFkB*, *TNFA* and *IL1B*) is generated against bacterial lipopolysaccharide (LPS) in the bovine oviduct^3,4^, while an anti-inflammatory response (*PTGES*, *TGFB1* and *IL10*) is enhanced in the presence of allogenic sperm *in vitro*^5^.

After fertilization, the developing embryo spends about 4 days in the bovine oviduct and then enters into the uterus at the 16-cell to early morula stage^6^. In a recent study, it was necessary to transfer 50 zygotes into the oviduct to detect differences in the oviduct transcriptome on D3 (D0 = estrus) of pregnancy in heifers, while single embryo transfers did not induce detectable changes in gene expression, suggesting that a very local and weak effect of the embryo exists in the oviduct^7^. The same group also observed transcriptional differences between ampulla and isthmus regions of the bovine oviduct, when a D3 embryo was present^8^. These findings suggest that embryo-maternal communication starts in the oviduct in the cow. Nevertheless, the molecular mechanism by which the embryo communicates with the bovine oviduct has not been well characterized. It was observed that early cleavage-stage bovine embryos express *MHC I* transcript^9^ and thus they could be detected as foreign by the oviduct immune system. Therefore, it is possible that the immunological crosstalk between the embryo and mother starts in the oviduct in the cow.

Interferon-tau (IFNT), initially named as trophoblastin, is a trophoectoderm-derived cytokine responsible for the process of maternal recognition of pregnancy in ruminants^10–13^. IFNT is also regarded as an immunosuppressive molecule that inhibits lymphocytes proliferation and therefore may play an important role for protection of the semi-allogenic embryo from attack by maternal immune system^14^. Recently, we demonstrated *in vitro* that D5-D9 bovine embryos starts to produce IFNT, which acts as one of the major players for generation of an anti-inflammatory response in the uterus^15^. IFNT has been shown to stimulate the expression of interferon-stimulated genes (*ISGs*), not only in the uterus^16–18^, but also in the corpus luteum^19^ and peripheral blood leukocytes^20,21^ during early pregnancy in cows. To the best of our knowledge, there is no information on the involvement of IFNT in embryo-maternal communication in the bovine oviduct. It was demonstrated that *IFNT* mRNA is expressed in the 8 to16-cell bovine embryo *in vitro*^22,23^, suggesting that the early embryo starts to produce IFNT in the bovine oviduct; however, this has not been investigated. We hypothesized that IFNT from 8 to16-cell stage embryos modulates the “local” immune response in the bovine oviduct. Thus, we investigated the effect of pre-blastocyst embryos on immune-related gene profile in bovine oviduct epithelial cells (BOECs) *in vitro*, and also examined the effect of conditioned media from embryo alone cultures and embryo-BOEC co-cultures on gene expression of peripheral blood mononuclear cells (PBMCs).

## Results

### Specificity of anti-IFNT antibody

**Figure 1:**
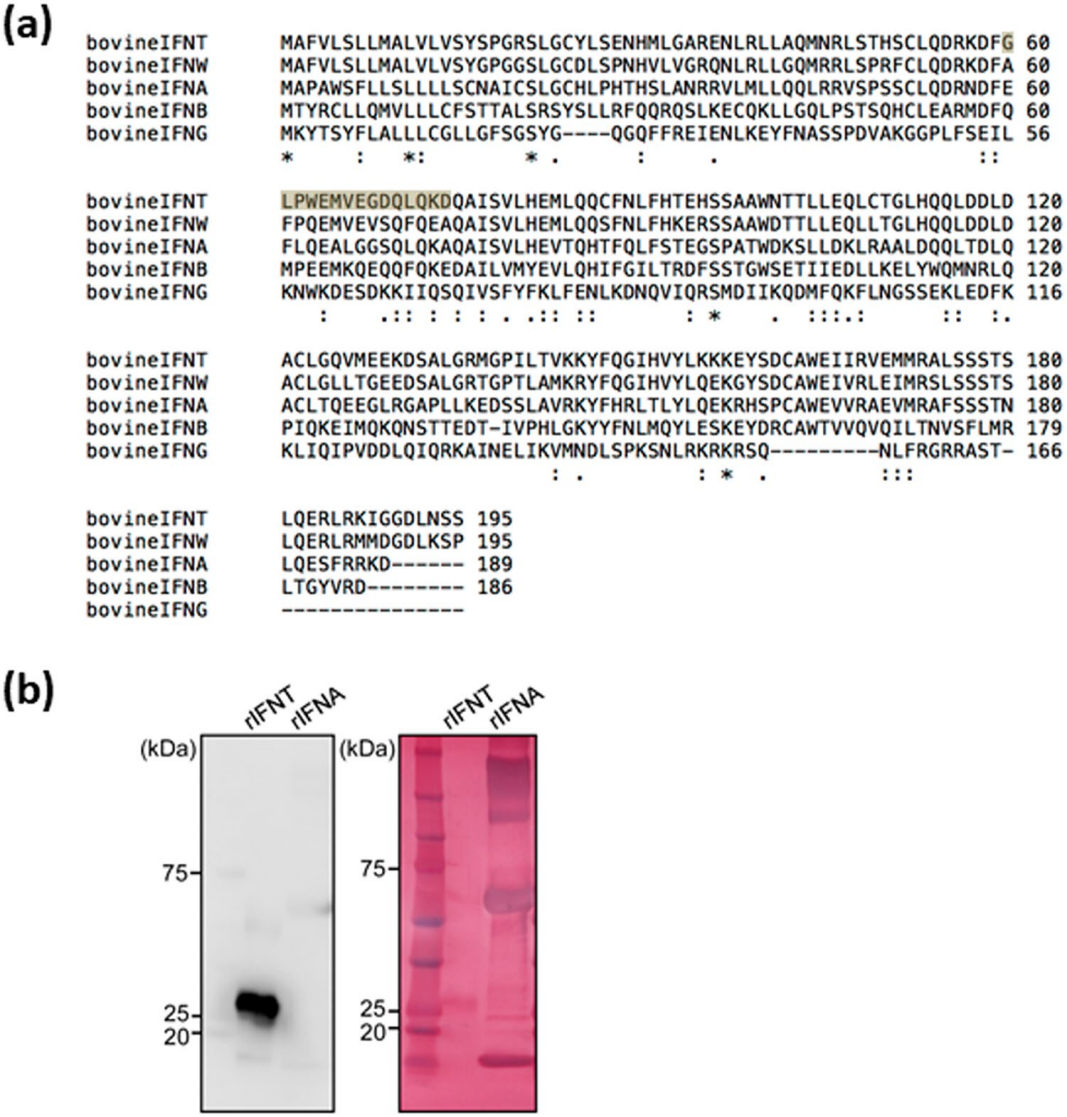
(**a**) Comparison of the amino acid sequences of bovine IFNT, IFNW, IFNA, IFNB, and IFNG. The gray region represents the target of anti-IFNT antibody. (**b**) Left: recombinant IFNT, but not recombinant IFNA was detected using anti-IFNT antibody. Right: the PVDF membrane was stained with Colloidal Gold Total Protein Stain solution to confirm total protein loading. It showed bands at the molecular masses of the recombinant proteins. Full-length blots are presented in Supplementary Figure 5.

An antibody to bovine IFNT (NP_001026935.1) was used to detect IFNT production by bovine embryos. The antibody was produced using the peptide specific for bovine IFNT (C + GLPWEMVEGDQLQKD), but not for bovine interferon-omega (bIFNW, NP_776776.1), interferon-alpha (bIFNA, NP_001017411.1), interferon-beta (bIFNB, NP_776775.1), or interferon-gamma (bIFNG, NP_776511.1) (Fig. 1a).

To confirm the specificity of anti-IFNT antibody, recombinant bovine IFNT and IFNA were subjected to PAGE gel electrophoresis, followed by western blot analysis. Recombinant IFNT was detected using this anti-IFNT antibody (Fig. 1b).

### IFNT was detected in embryos co-cultured with BOECs

**Figure 2:**
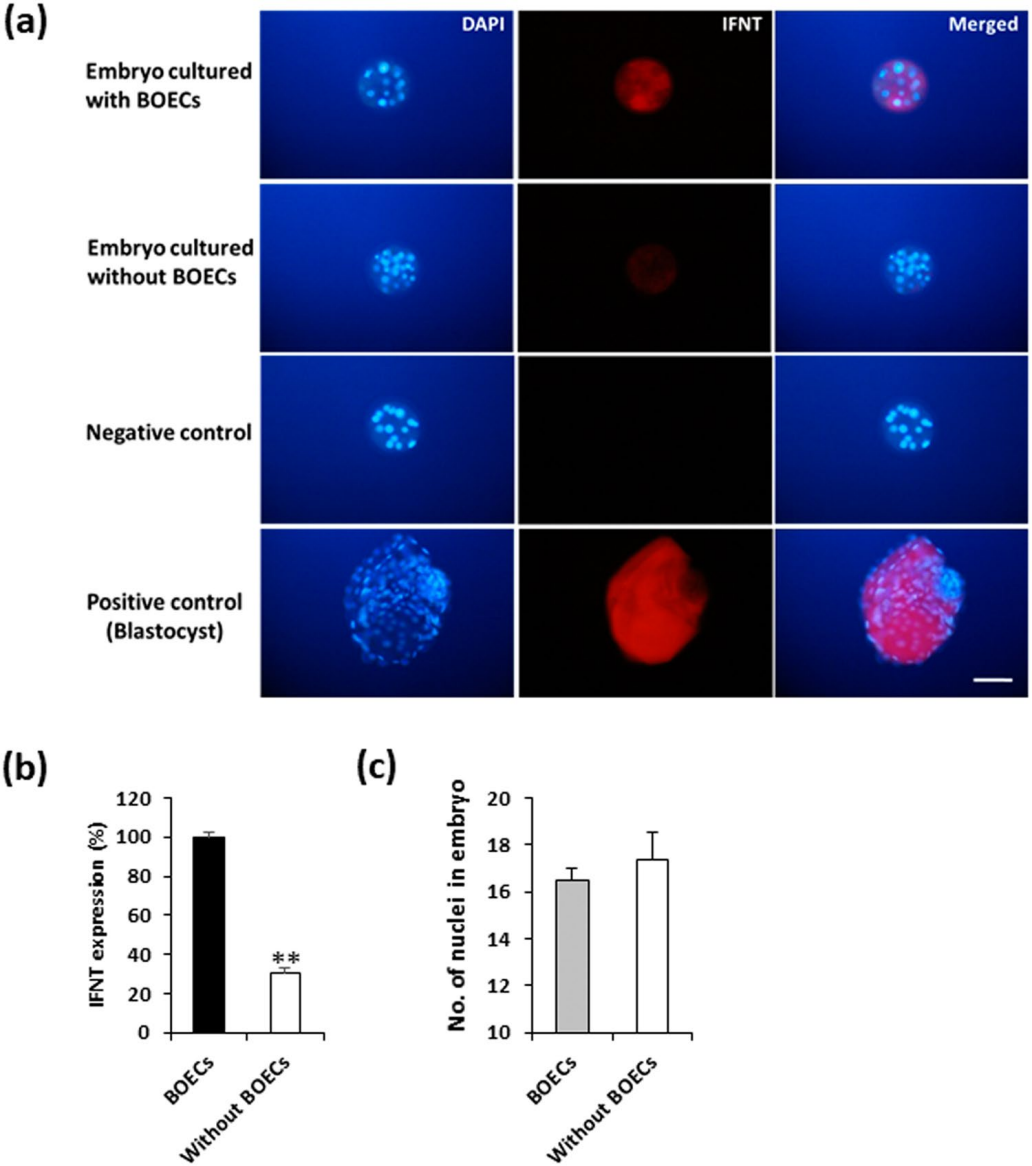
(**a**) IFNT expression in the developing early morulae after culture with and without BOECs. Embryos stained without the primary antibody for IFNT served as a negative control. IFNT expression in blastocyst served as a positive control. Scale bar = 100 µm. (**b**) Semi-quantitative analysis of IFNT protein expression in embryos cultured in the presence or absence of BOECs. (**c**) Mean ± SEM number of nuclei in early morulae cultured with and without BOECs. Number of embryos examined from each group = 19. Data are presented as mean ± SEM. **P < 0.01 denote significant difference.

When embryos were co-cultured with BOECs, IFNT was detected in the early morulae (at 16-cell stage) on D4; whereas IFNT was not detected in the embryos cultured without BOECs (Fig. 2a,b). IFNT was not detected in the embryos co-cultured with BOECs at 2-cell, 4-cell or 8-cell stages (data not shown). The number of cells was similar in developing morulae cultured with or without BOECs (Fig. 2c).

### Co-culture with embryos affected gene expression and PGE2 secretion in BOECs

**Figure 3:**
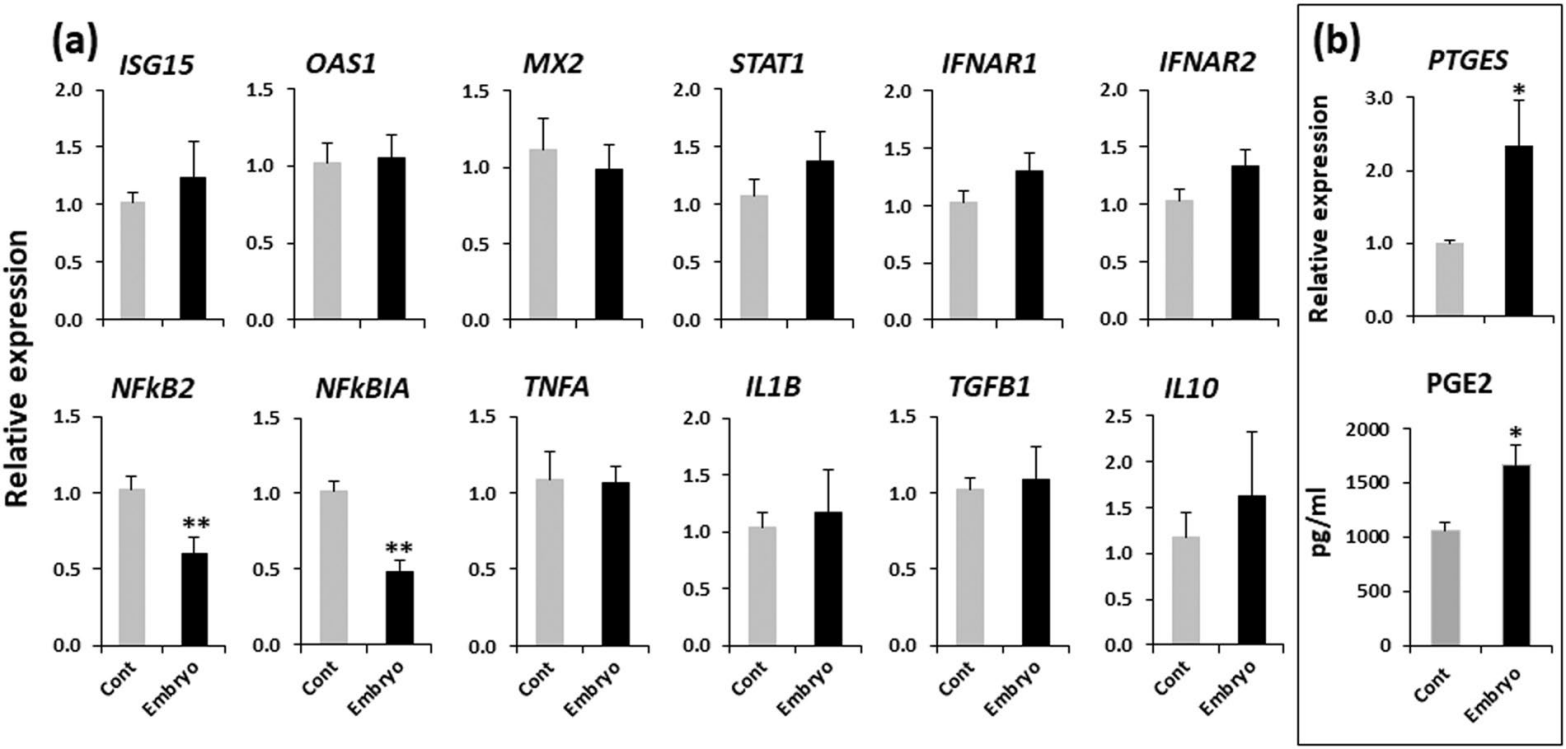
(**a**) Relative mRNA expression of candidate genes in BOECs cultured with and without (cont) embryos. (**b**) Relative mRNA expression of *PTGES* and PGE2 secretion in BOECs cultured with and without (cont) embryos. Data are presented as mean ± SEM of six independent experiments performed in duplicate. Three to four oviducts from three to four different cows were used for BOECs culture in each experiment. *P < 0.05, **P < 0.01 denote significant difference.

Co-culture of embryos with BOECs significantly suppressed *NFkB2* and *NFkBIA* (transcription factors for inflammatory and immune response) expression (*P* < 0.01), and stimulated *PTGES* (an enzyme related to PGE2 synthesis) expression and PGE2 secretion (*P* < 0.05) in BOECs, indicating that an anti-inflammatory response to embryos was generated in the oviduct epithelium. However, pro- (*TNFA* and *IL1B*) and anti- (*TGFB1* and *IL10*) inflammatory cytokine expression in BOECs did not differ in the absence or presence of embryos. Also, the expression of interferon (IFN)-stimulated genes (*ISGs*; *ISG15*, *OAS1* and *MX2*), a transcription factor for IFN-signaling (*STAT1*), or type-1 IFN receptors (*IFNAR1* and *IFNAR2*) in BOECs was not influenced by the presence of embryos (Fig. 3). Together, these results indicate that a crosstalk between the embryos and BOECs exists, but this was not mediated by IFNT.

### Medium from embryo-BOEC co-cultures stimulated gene expression in PBMCs

**Figure 4:**
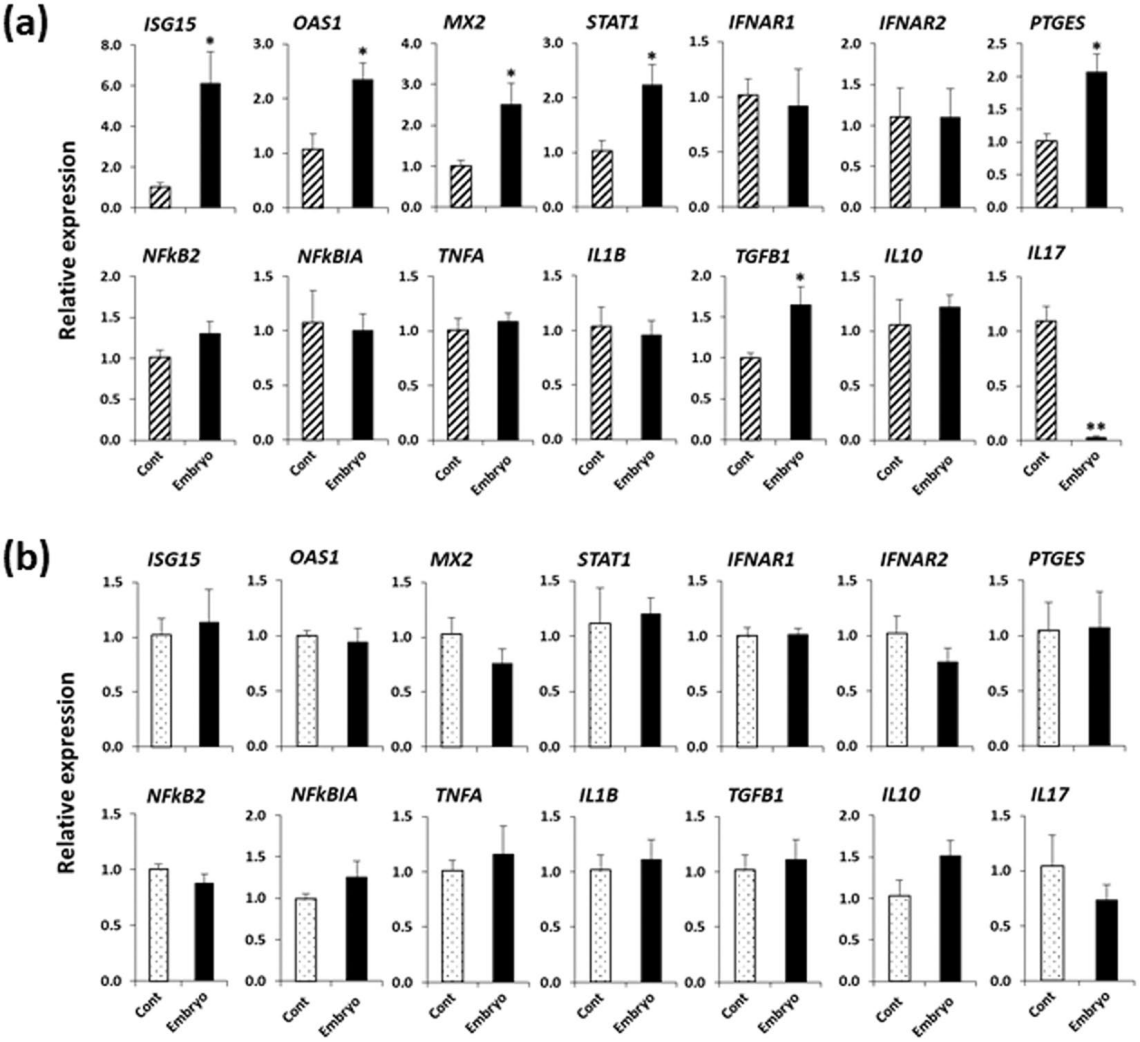
(**a**) Relative mRNA expression of candidate genes in PBMCs cultured in embryo-BOEC co-cultures medium or in BOEC cultures medium (cont). (**b**) Relative mRNA expression of candidate genes in PBMCs cultured in medium from embryos alone cultures or in culture medium without embryos (cont). Data are presented as mean ± SEM of six independent experiments performed in duplicate. *P < 0.05, **P < 0.01 denote significant difference.

Medium collected from embryo-BOEC co-cultures stimulated *ISGs* and *STAT1* in PBMCs (*P* < 0.05). Moreover, this co-culture medium significantly increased *PTGES* and *TGFB1* expression, but suppressed *IL17* expression in PBMCs (*P* < 0.05) (Fig. 4a). On the other hand, the medium from culture of embryos alone did not affect *ISGs* or other immune-related genes in PBMCs (Fig. 4b).

### IFNT-treated BOEC culture media and IFNT-supplemented fresh media alone without BOEC both affected gene expression in PBMCs

**Figure 5:**
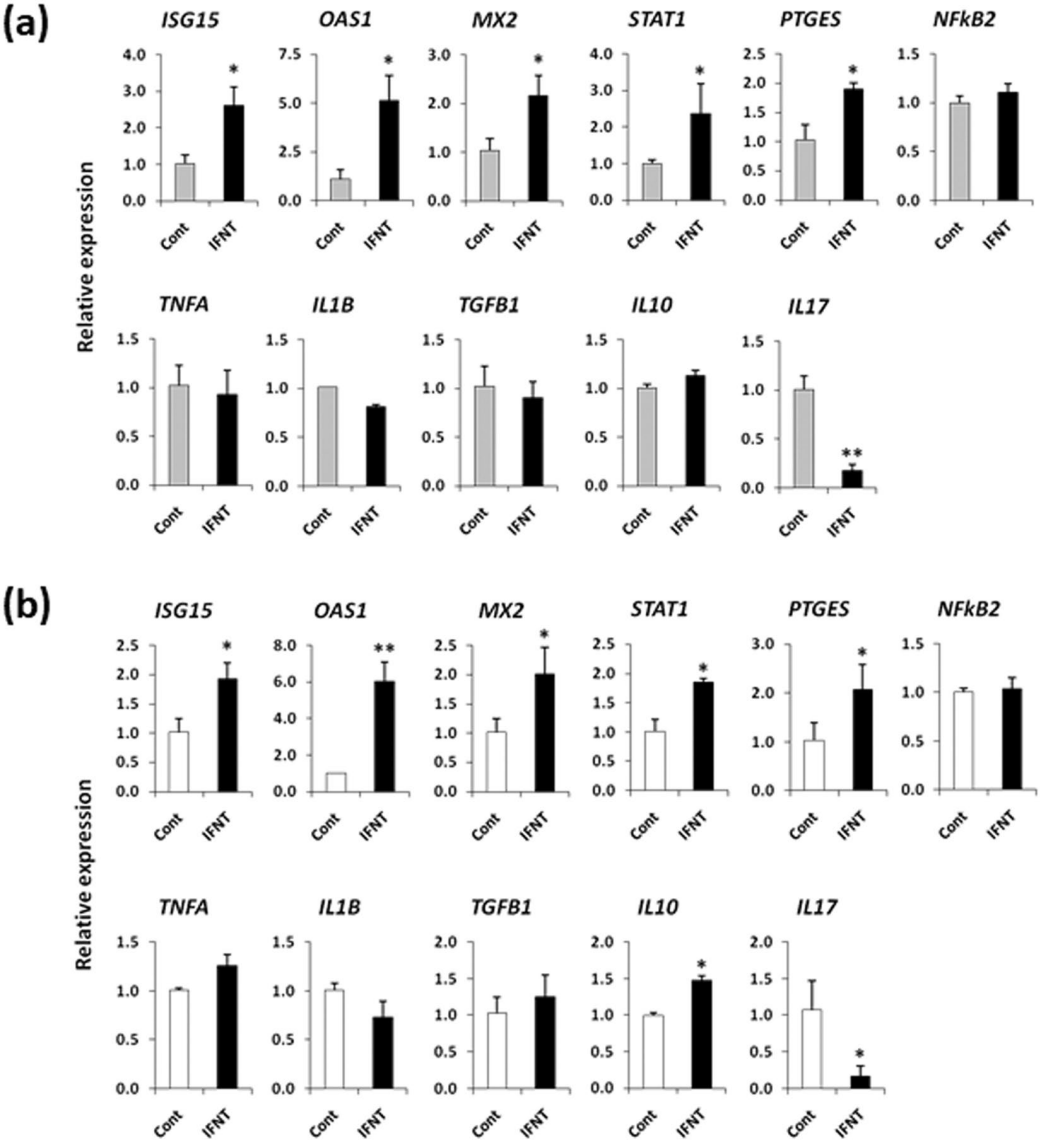
(**a**) Relative mRNA expression of candidate genes in PBMCs cultured in IFNT-treated (50 pg/ml) BOEC medium or in BOEC medium (cont). (**b**) Relative mRNA expression of candidate genes in PBMCs cultured in fresh medium supplemented with IFNT (50 pg/ml) or in fresh medium without IFNT (cont). Data are presented as mean ± SEM of three independent experiments performed in triplicate. *P < 0.05, **P < 0.01 denote significant difference.

Similar to the embryo-BOEC medium, IFNT (50 pg/ml)-treated BOEC medium stimulated *ISGs* (*ISG15*, *OAS1*, *MX2*), *STAT1*, *PTGES* (*P* < 0.05), and suppressed *IL17* in PBMCs (*P* < 0.01) (Fig. 5a). However, *TGFB1* was not up-regulated. Likewise, IFNT (50 pg/ml)-supplemented fresh medium alone without BOECs stimulated *ISGs*, *PTGES* and decreased *IL17* in PBMCs (Fig. 5b). In order to test the sensitivity of BOECs to IFNT, BOECs were treated with 25, 50, or 100 pg/ml of IFNT for 24 h. It was found that 100 pg/ml IFNT was required to stimulate *ISGs* in BOECs (Supplementary Figure 1a), which indicates that the concentration of IFNT in embryo-BOEC co-culture medium was less than 100 pg/ml.

### Antibody neutralization of IFNT in embryo-BOEC co-cultures blocked changes in gene expression in PBMCs

**Figure 6:**
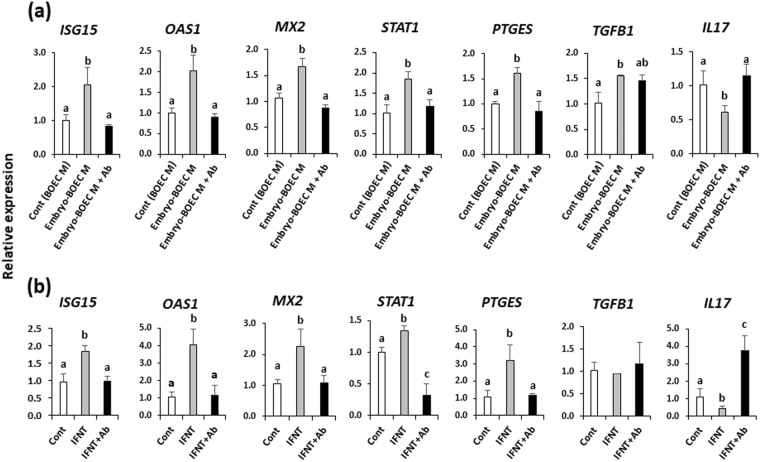
(**a**) Relative mRNA expression of target genes in PBMCs cultured in embryo-BOEC co-culture medium after antibody neutralization of IFNT. (**b**) Relative mRNA expression of target genes in PBMCs after antibody neutralization of IFNT (50 pg/ml). Data are presented as mean ± SEM of three independent experiments performed in triplicate. Different letters (a,b,c) above the bars for each gene denote significant differences, when compared to the control (cont).

The media from embryo-BOEC co-cultures did not increase *ISGs* (*ISG15*, *OAS1*, *MX2*) and *STAT1* expression after neutralization of IFNT using an anti-IFNT antibody (Ab), nor did it stimulate *PTGES* or suppress *IL17* (Fig. 6a). Adding anti-IFNT antibody to IFNT (50 pg/ml) before addition to cultures blocked effects of IFNT on PBMCs (Fig. 6b).

## Discussion

Our results show, for the first time, that bovine D4 embryos at the 16-cell stage express IFNT when cultured with oviduct epithelial cells. We provide *in vitro* evidence that an immunological crosstalk indeed exists between the early embryo and BOECs. It should be noted that, in addition to *ISGs* activation, the conditioned medium from embryo-BOEC co-cultures generated an anti-inflammatory response in immune cells (PBMCs). Our evidence strongly suggests that a very small amount of IFNT produced by the developing embryo in the oviduct is likely to be involved in modulating gene expression in the immune cells. In a previous study from this laboratory, a considerable number of immune cells were found in the cow oviduct under physiological condition, where polymorphonuclear neutrophils (PMNs) constituted ∼17% and lymphocytes represented ∼23% of the total leukocyte count^24^. It is also reported that macrophages/dendritic cells and lymphocytes are present in the bovine oviduct mucosa^25,26^. Until now, there has been no information on how local immune cells respond to a semi-allogenic embryo in the bovine oviduct. Since the number of immune cells in the oviduct mucosa is limited and their isolation from the oviduct is very difficult, this has not yet been studied. PBMCs are composed of mainly lymphocytes and some monocytes. Therefore, responses of immune cells (PBMCs) after incubation with embryo-BOEC co-cultures media in this study might represent the possible interaction between the embryo and immune cells in the bovine oviduct.

Our findings indicate that pre-blastocyst embryos interact with oviduct epithelial cells and also can communicate with local immune cells present in the bovine oviduct. Thus, the *in vitro* co-culture of early embryos with BOECs and further the impact of embryo-BOECs co-culture medium on immune cell (PBMCs) gene expression could provide a valuable tool to investigate the molecular mechanisms and regulatory pathways involved in embryo-oviduct interaction in cattle. One could argue that we used multiple embryos (25–30 zygotes) for co-culture, which does not naturally happen *in vivo* in cattle. In our study, we used multiple embryos in order to amplify the signals from embryos, such that changes in gene expression in BOECs and also in PBMCs could be clearly detected. In fact, multiple embryos are known to develop normally in the oviduct and uterus of superovulated cattle until D7 of pregnancy^27^. Therefore, it is likely that the multiple embryos in our study did not elicit abnormal responses in BOECs and PBMCs gene expression. It should be noted that multiple embryos have been used by others to study embryo-oviduct interactions in cattle *in vitro* and *in vivo*^7,28^.

In this study, the D4 embryos expressed IFNT after co-culture with BOECs; however, IFNT was not expressed in the embryos when cultured alone for 4 days. This finding suggests that physical contact and/or some factors from BOECs stimulate early cleavage-stage embryos to synthesize IFNT. In our experimental model, we did not culture embryos with non-oviduct epithelial cell feeders (for example VERO cells or fibroblasts) as various controls to clarify the specific impact of oviduct on IFNT synthesis by D4 bovine embryos, which requires further investigation. It is well established that the oviduct supports the physiological events-related to fertilization, cleavage process and pre-implantation embryo development by providing various factors. This phenomenon was first described in the sheep^29^, where the embryos were cultured with oviduct epithelial cells or fibroblasts. Gandolfi and Moor^29^ found that 80% of embryos grown on oviduct epithelial cells, whereas only 33% of embryos grown on fibroblasts, were fully viable as evaluated by their ability to develop after transfer to recipient sheep. They concluded that cleavage occurs at the same rate on different feeder-layers, but oviduct epithelial cells appear to be required for the acquisition of full embryonic viability. This finding indicates that the presence of oviduct epithelial cells is essential for pre-implantation embryo development and viability, which is not achieved by any type of somatic cell. Subsequently, the enhancement of embryo development after co-culturing with oviduct epithelial cells or oviductal tissue has also been demonstrated in cattle^30^. Importantly, it has been reported that FGF2 or EGF enhance secretion of IFNT by bovine trophoblasts *in utero*^31,32^. A variety of factors, including FGF2 and EGF, have been identified to play important roles in the bovine oviduct^33,34^. Therefore, it is likely that these factors from oviduct epithelium might be involved in synthesis of IFNT by early embryos. In our study, although the embryos expressed IFNT in the presence of BOECs, *ISGs* expression was not stimulated in BOECs. In contrast, another group reported that early bovine embryos can stimulate *ISGs* in BOECs after 8 days of co-culture, where embryos had developed to the blastocyst stage^28^. Since bovine embryos normally reach the blastocyst stage after they have entered the uterus, the expression of *ISGs* in BOECs in that study did not represent an oviduct-specific response that would occur *in vivo*.

Our study revealed that embryos stimulate *PTGES* expression and PGE2 secretion in BOECs. PGE2 was detected in *in vitro*-produced bovine embryos by immunofluorescence staining, while intensity of PGE2 staining gradually increased from 2-cell to blastocyst^35^. Therefore, it is likely that both the embryo and oviduct epithelial cells contribute to generate a PGE2 rich environment in the bovine oviduct. PGE2 is known as an immunosuppressive molecule that inhibits lymphocytes proliferation in culture at a concentration of 10^−8^ M^36^. Our laboratory recently demonstrated that sperm stimulate oviduct epithelial cells to secrete PGE2, which acts as a key regulator for generation of anti-inflammatory responses in the bovine oviduct *in vitro*^5^. Moreover, a previous study from our laboratory showed that PGE2 plays a major role in suppressing sperm phagocytosis by PMNs in the bovine oviduct in a dose-dependent manner^24^. Now we have shown that embryo-stimulated PGE2 biosynthesis in BOECs appears to be involved in suppression of local immunity in the oviduct. The embryos generated an anti-inflammatory response in BOECs *via* suppression of *NFkB2* and *NFkBIA*, key factors for initiation of inflammatory and immune responses. A similar result was also observed by Maillo *et al*.^7^ who demonstrated that transfer of 50 embryos into the oviduct down-regulated *NFkB2* expression in cattle.

In the present study, the mechanism by which PGE2 secretion was increased in BOECs by the embryos is not known; however, the medium conditioned by embryo-BOEC co-culture stimulated the expression of *PTGES* and *TGFB1*, with activation of *ISGs* in PBMCs. It is well established that TGFB works synergistically with PGE2 to enhance the differentiation of naïve T cells (Th0) to regulatory T cells for immune tolerance^37^. On the other hand, TGFB also promotes the differentiation of Th0 to Th17 cells, which express *IL17*, in the presence of pro-inflammatory cytokines (IL1 or IL6)^38,39^. In our study, expression level of *IL17* was decreased in PBMCs by embryo-BOEC co-cultures medium. Therefore, embryo-stimulated *TGFB1* in PBMCs and PGE2 synthesis from BOECs may act synergistically to generate anti-inflammatory and immune tolerance conditions in the bovine oviduct. We cannot exclude the possibility that immune responses of the oviduct to a semi-allogenic embryo or allogenic embryo could vary. In this study, embryos are indeed allogenic to BOECs monolayer, since the oocytes for embryo production and BOECs for co-culture with embryos were collected from different cows. This does not happen in the cow following insemination, where oocytes are fertilized by the sperms and resultant embryo develops in the oviduct and uterus, thus embryo is semi-allogenic to the mother.

In accordance with IFNT expression in D4 embryos co-cultured with BOECs, embryo-BOEC co-culture medium increased *ISGs* and *STAT1* in PBMCs, suggesting that our co-culture medium contained IFNT, even though the embryos did not stimulate *ISGs* in BOECs. The possible reason is that PBMCs are much more responsive to IFNT than are BOECs, because they expressed more type-1 IFN receptors than BOECs (Supplementary Figure 2). Unfortunately, we were not able to determine concentration of IFNT in the medium from embryo-BOEC co-cultures. This could be due to the presence of 5% FCS in the culture medium, which appears to disrupt the sensitivity and specificity of ELISA. Of note, the medium from embryo culture alone did not stimulate *ISGs* or other immune-related genes in PBMCs, suggesting that the change of gene expression in PBMCs is IFNT-dependent, and that this medium contained no IFNT or the amount was less than that present in embryo-BOEC co-culture medium.

Interestingly, IFNT at 50 pg/ml did not activate *ISGs* in BOECs, nor did it suppress *NFkB2* or stimulate *PTGES* (data not shown) as did embryos. It appeared that changes in BOECs gene expression are regulated by embryo-derived factors other than IFNT. On the other hand, IFNT (50 pg/ml)-treated BOEC medium increased *ISGs* in PBMCs with stimulation of *PTGES* and suppression of *IL17*, similar to embryo-BOEC medium. However, like embryo-BOEC medium, IFNT-treated BOEC medium did not stimulate *TGFB1* in PBMCs. IFNT (50 pg/ml) alone without BOEC medium also induced similar changes of gene expression in PBMCs like embryo-BOEC medium and IFNT (50 pg/ml)-treated BOEC medium, suggesting that regulation of immune cells gene expression is IFNT-dependent and BOEC medium has no role in modulating PBMCs gene expression. Importantly; antibody neutralization of IFNT in the embryo-BOEC medium diminished the changes of gene expressions in PBMCs. Together, these results clearly indicate the involvement of IFNT, as one of the major players from the embryo, in the regulation of gene expression in PBMCs. This finding is in agreement with our recent study, in which IFNT from D5-D9 embryos promotes an anti-inflammatory response in uterine epithelial cells and immune cells^15^. In our study, other factors derived from the early embryo might also be involved in this immunological interaction between the embryo and immune cells. It has been proposed that the pre-hatching embryo (at least in humans and mice) produces certain soluble factors that are recognized by local immune cells in the reproductive tract, and cause tolerance of the maternal immune system to accept the embryo during the very early stages of pregnancy^40,41^.

**Figure 7:**
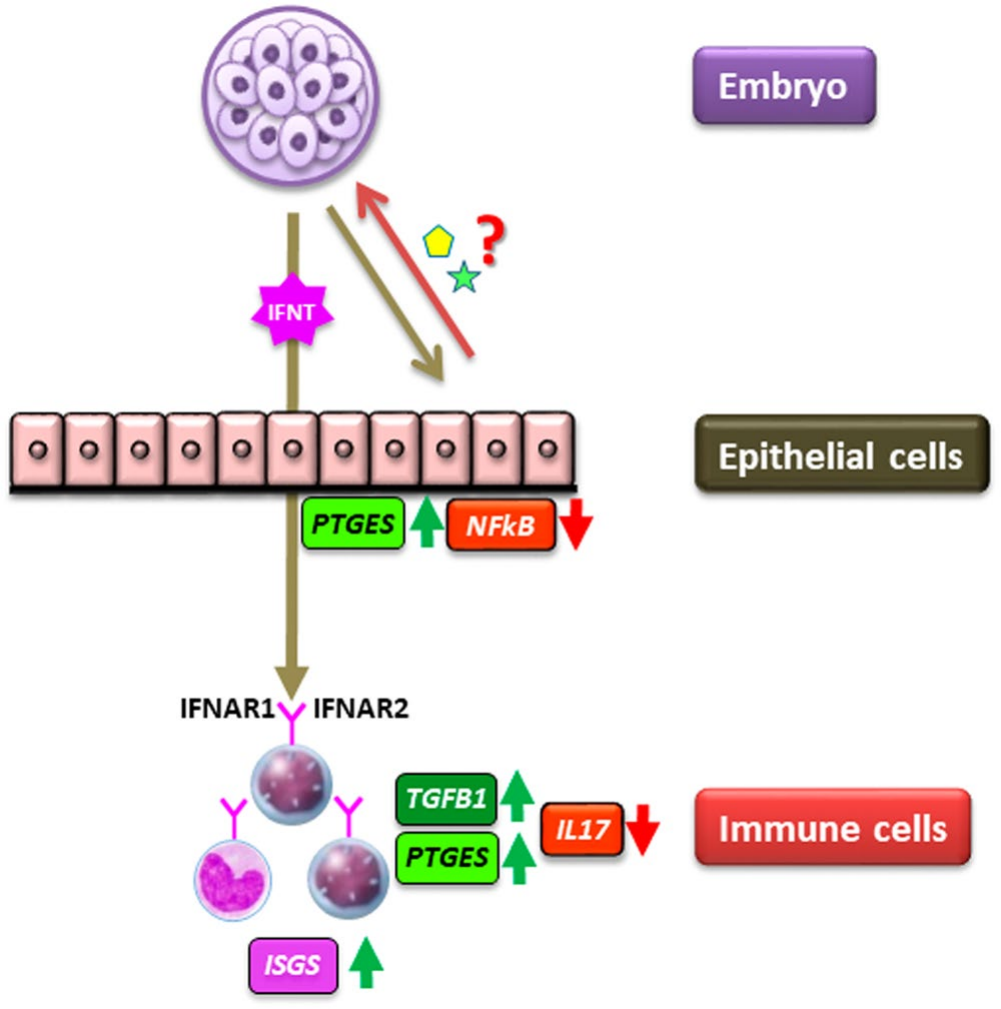
Our proposed model of the communication of the developing embryo with epithelium and immune cells in the cow oviduct. First, an immunological crosstalk exists between the embryo and oviduct epithelium; where the embryo generates an anti-inflammatory response in oviduct epithelium, and the oviduct epithelium helps the embryo to secrete IFNT by either physical contact and/or *via* releasing some factors. The embryo-derived IFNT then regulates gene expression of immune cells towards an anti-inflammatory response.

In conclusion, findings of the present study support the hypothesis (Fig. 7) that the bovine embryo at the morula stage starts to secrete IFNT in the oviduct with the help of stimulation from oviduct epithelial cells, and that the embryo stimulates an anti-inflammatory response in BOECs without *ISGs* activation. The interaction between the embryo and BOECs generates an anti-inflammatory response in immune cells with stimulation of *ISGs*. IFNT from developing bovine embryo is likely to be involved in regulation of such a “local” immune response in the oviduct, which could enable the morula to survive and pass on to the uterus. Further study is needed to investigate gene expressions in “local” oviduct epithelium and immune cells in the presence of an embryo on D4 after insemination in cows, including the impact of *(i)* follicular fluid that enters into the oviduct at the time of ovulation, *(ii)* sperm binding to oviduct epithelium following insemination and fertilization process itself, and *(iii)* endocrine changes that result from ovulation.

## Methods

### Ethics statement

The Committee on the Ethics of Animal Experiments of the Obihiro University of Agriculture and Veterinary Medicine approved the protocol (Permit number 25–101). Blood was collected from the cow in accordance with the Guiding Principles for the Care and Use of Research Animals promulgated by Obihiro University of Agriculture and Veterinary Medicine, Japan.

### Experimental model

First, BOECs were co-cultured with IVF-derived zygotes (n = 25–30) for 4 days, which mimics the embryo development in the bovine oviduct *in vivo*. At the end of co-culture, 10–15 embryos had developed to the 16-cell stage (early morula). As a control, BOECs were also cultured without embryos. The embryos were examined for IFNT by immunofluorescence labelling, and analysis of BOEC gene expression was performed. Subsequently, PBMCs were cultured in conditioned from embryo-BOEC co-cultures or BOEC cultures alone (control) and gene expression was analyzed. Next, zygotes (n = 25–30) were cultured alone without BOECs for 4 days, where 10–12 embryos had developed to around the 16-cell stage. The embryo culture media were then collected and embryos were immunolabelled for IFNT. At the same time, fresh medium was incubated without embryos for 4 days. Further, PBMCs were cultured in conditioned medium from embryo alone culture or in culture medium without embryos (control) for analysis of gene expression (Supplementary Figure 3).

### Primary culture of BOECs

The oviducts were collected from a local abattoir (Obihiro, Hokkaido, Japan). The phase of estrous cycle was determined as reported earlier^42^. Only healthy oviducts ipsi-lateral to the corpus luteum during early luteal phase (D4–5) were used in this study. Oviducts were ligated at both ends and then immersed in phosphate buffer saline without calcium or magnesium (PBS−/−), supplemented with 1% penicillin-streptomycin (Gibco, Grand Island, USA) and 1% amphotericin B (Gibco), and then transported to the laboratory on ice. In the laboratory, oviducts were separated from the surrounding connective tissue, rapidly rinsed in 70% ethanol for disinfection, and rinsed three times with PBS−/−. In total, three to four oviducts from three to four different cows were used in each experiment. Oviduct epithelial cells were isolated and cultured as previously described^43^ with slight modifications. Briefly, BOECs were cultured in medium containing DMEM/F12 (Gibco) supplemented with 2.2% NaHCO_3_ (Sigma-Aldrich, Steinheim, Germany), 1% penicillin-streptomycin, 1% amphotericin B, and 10% fetal calf serum (FCS; Bio Whittaker, Walkersville, MD) in 4-well culture plates (Nalge Nunc International, Roskilde, Denmark) at 38.5 °C under a humidified atmosphere of 5% CO_2_ in air. The culture medium was renewed every alternate day until the BOECs attained 80–90% confluence, when they were used for co-culture with the embryos. The cells in culture medium showed the characteristics of cultured monolayers of epithelium, which was further confirmed by immunofluorescence labelling using a monoclonal antibody against cytokeratin (anti-cytokeratin 8 + 18; ab53280, Abcam, Tokyo, Japan) according to a protocol described previously^44^. The purity of cultured BOECs was >98% (Supplementary Figure 4a,b).

### *In vitro* embryo production

Bovine ovaries were collected from a local abattoir (Obihiro, Hokkaido, Japan) and then transported to the laboratory in normal saline (0.85% NaCl) supplemented with 1% penicillin-streptomycin at 38.5 °C within 1‒2 h of slaughter. The *in vitro* maturation (IVM) and fertilization (IVF) were performed as described earlier^45^. Cumulus oocyte complexes (COCs) with a homogenous cytoplasm and surrounded by at least three layers of compact cumulus cells were matured *in vitro* for 22 h in 4-well plates, in groups of 30–40 per well in 500 µl HP-M199 medium (Research Institute for the Functional Peptides Co., Ltd.) supplemented with 10 µg/ml FSH (Folltropin-V, Bioniche Animal Health Inc., Belleville, Ontario, Canada) and 5% FCS at 38.5 °C under an atmosphere of 5% CO_2_ in humidified air. *In vitro*-matured COCs (n = 15) and sperm (5 × 10^6^/ml) were co-incubated for 6 h in droplets containing 50 µl medium IVF100 (Research Institute for Functional Peptides Co., Ltd.) at 38.5 °C under an atmosphere of 5% CO_2_ in humidified air. Next, the presumptive zygotes were denuded by vortex and cultured *in vitro* (IVC) in groups of 15 in 50 µl droplets of KSOMaa medium (Zenith Biotech, Guilford CT, USA) supplemented with 5% FCS under mineral oil at 38.5 °C under an atmosphere of 5% O_2_, 5% CO_2_ and 90% N_2_ in humidified air. After 12 h of IVC (18 h post-IVF), presumptive zygotes were transferred onto BOEC monolayers for co-culture experiments.

To produce embryo culture medium without epithelial cells (Supplementary Figure 3), zygotes were cultured for 4 days in 4-well plates, in groups of 25–30 in 400 µl KSOMaa supplemented with 5% FCS without mineral oil. At the end of culture, embryos were removed and medium was collected (denoted as embryo alone culture medium).

### Embryo-BOEC co-culture

Approximately, 6 h before transferring the zygotes, BOECs culture medium was entirely replaced with equal amount of KSOMaa medium (400 µl) supplemented with 5% FCS. The presumptive zygotes, approximately18 h post- IVF, were transferred onto BOEC monolayers in 4-well plates, in groups of 25–30 per well and cultured for 4 days at 38.5 °C under a humidified atmosphere of 5% O_2_, 5% CO_2_ and 90% N_2_ in air. At 48 h co-culture, half of the medium was replaced. At the end of co-culture, supernatants were collected and stored at −80 °C for ELISA and immune cell experiments. BOECs were lysed with Trizol (Invitrogen, Carlsbad, USA) and stored at −80 °C for RNA extraction. The early morulae, developed on the BOEC monolayer, were examined by immunofluorescence labelling. The co-culture experiments were repeated six times and 45.1 ± 2.9% (n = 110/248) embryos had developed into early morulae (≥16-cell) after 4 days co-culture (Supplementary Figure 4c,d).

### Isolation of PBMCs

PBMCs were isolated from whole blood according to protocol described previously^20^ with slight modifications. Heparinized blood from a multiparous Holstein cow during early luteal phase (D4–5) was collected and gently mixed with an equal volume of PBS−/−. The diluted blood was slowly layered over Ficoll-paque solution (Lymphoprep, Axis Shield, Oslo, Norway), and centrifuged at 10 °C for 35 min at 1000 × *g*. PBMCs were collected from buffy coat (white layer containing mononuclear cells) layer, then mixed with hemolysis buffer (NH_4_Cl 155 mM, KHCO_3_ 9.9 mM, EDTA 96.7 μM) for 10 sec, and centrifuged at 10 °C for 10 min at 500 × *g* to purify from red blood cells (RBCs). Subsequently, the cell pellet was washed twice by PBS−/−. The purity of collected PBMCs was >98% as evaluated by flow cytometry and the viability was about 99% as assessed by Trypan blue staining.

### Culture of PBMCs in the embryo-BOEC co-culture medium

PBMCs (5 × 10^6^ cells) were cultured in 400 µl embryo-BOEC co-culture medium or in BOEC culture medium without embryos (control) in a 48-well plate (Nalge Nunc International, Roskilde, Denmark) for 24 h at 38.5 °C under a humidified atmosphere in 5% CO_2_ in air. After culture, the supernatant was collected, and PBMCs were lysed using Trizol and stored at −80 °C for RNA extraction.

### Culture of PBMCs in the embryo culture medium

PBMCs (5 × 10^6^ cells) were cultured in 400 µl embryo alone culture medium or in culture medium that had been incubated without embryos (control) in a 48-well plate for 24 h at 38.5 °C under a humidified atmosphere of 5% CO_2_ in air. After culture, the supernatant was collected, and PBMCs were lysed using Trizol and stored at −80 °C for RNA extraction.

### Treatment of BOECs and PBMCs with recombinant bovine IFNT (bIFNT)

The BOEC monolayers were treated with 0 (control), 25, 50 or 100 pg/ml bIFNT (bIFNT2B, specific activity = 4.5 × 10^7^ U/mg; Zenoaq, Koriyama, Japan) for 24 h at 38.5 °C under a humidified atmosphere of 5% CO_2_ in air.

PBMCs (5 × 10^6^ cells) were cultured in IFNT-treated BOEC medium for 24 h at 38.5 °C under a humidified atmosphere of 5% CO_2_ in air. At the same time, PBMCs were also cultured directly in fresh medium containing 0 (control), 25, 50 or 100 pg/ml IFNT for 24 h at 38.5 °C under a humidified atmosphere of 5% CO_2_ in air. At the end of treatment, supernatant was collected, and the cells (BOECs and PBMCs) were lysed using Trizol and stored at −80 °C for RNA extraction.

The dose of IFNT (50 pg/ml) was selected on the basis of gene expression in BOECs and PBMCs, where *ISG15* was not activated in BOECs, but up-regulated in PBMCs after culture in fresh medium containing 50 pg/ml IFNT or in IFNT-treated BOEC medium, as with the embryos (Supplementary Figure 1).

### Production of anti-bovine IFNT antibody

Production of rabbit polyclonal anti-bovine IFNT antibody was carried out at Eurofins Genomics (Tokyo, Japan). The antibody was produced using the peptide specific for bovine IFNT (C + GLPWEMVEGDQLQKD), but not for bovine interferon-omega (bIFNW, NP_776776.1), interferon-alpha (bIFNA, NP_001017411.1), interferon-beta (bIFNB, NP_776775.1), or interferon-gamma (bIFNG, NP_776511.1).

### Western blotting to confirm specificity of anti-IFNT antibody

Recombinant bovine interferon-tau (IFNT) or interferon-alpha (IFNA) (200 ng)^46^ were mixed with Laemmil 2× buffer (4% SDS, 10% 2-mercaptoethanol, 20% glycerol, 0.0% bromophenol blue, and 0.125 M Tris-HCl), and then boiled for 4 min. Samples were separated by SDS-PAGE and electrophoretically transferred to a polyvinylidene difluoride (PVDF) membrane (Millipore, Billerica, MA, USA). This membrane was blocked with Block ACE (DS Pharma Biomedical, Osaka, Japan), and probed with primary antibodies against IFNT (0.3 µg/ml, Eurofins Genomics, Tokyo, Japan). Immunoreactive bands were detected using enhanced chemiluminescence (Millipore) after incubation with horseradish peroxidase-labeled goat anti-rabbit IgG antibody (0.5 µg/ml, Vector Laboratories, Burlingame, CA, USA). Total proteins were stained with Colloidal Gold Total Protein Stain solution, according to the manufacturer’s instructions (Bio-Rad Laboratories, Hercules, CA, USA).

### Immunofluorescence labelling of the embryos for IFNT

The embryos, developed with and without BOECs, were examined for expression of IFNT protein by immunofluorescence labelling. First, the embryos were washed three times in PBS−/− containing 0.1% polyvinyl alcohol (PBS-PVA) and then fixed in 4% paraformaldehyde (PFA, Wako Pure Chemicals Ltd., Osaka, Japan) at 4 °C for 30 min. The embryos were exposed to 0.1% Tween-20 (Wako Pure Chemicals Ltd.) in PBS-PVA at room temperature for 30 min for permeabilization. Next, the embryos were incubated in 5% BSA in PBS at room temperature for 2 h. After blocking, the embryos were incubated with specific primary antibody (rabbit polyclonal anti-IFNT antibody, Eurofins Genomics, Tokyo, Japan; 1:200) at 4 °C for overnight. After incubation, the embryos were incubated again with secondary antibody (goat anti-rabbit IgG labelled with Alexa Flour 546, Invitrogen; 1:200) at room temperature for 2 h. Finally, the embryos were mounted with VECTASHIELD antifade mounting medium with DAPI (Vector Laboratories Inc., Canada) on glass slides. Images were captured using a fluorescence microscope (Nikon Microphot-SA, Tokyo, Japan), and ImageJ software was used to determine intensity of color.

### Neutralization of IFNT in the embryo-BOEC co-culture medium using anti-IFNT antibody

Neutralization experiments were performed to determine whether IFNT regulates PBMCs gene expression. First, embryo-BOEC co-culture medium was incubated in a 48-well plate with anti-IFNT antibody (1:300) for 1 h at 38.5 °C under a humidified atmosphere of 5% CO_2_ in air. Then, PBMCs (5 × 10^6^ cells) were added to that medium and cultured for 24 h. PBMCs were cultured in BOEC medium (control), and in embryo-BOEC co-culture medium without anti-IFNT antibody as a positive control. At the same time, additional experiments were performed using the same protocol to block the action of known IFNT (50 pg/ml). At the end of experiments, supernatant was collected and PBMCs were lysed using Trizol and stored at -80 °C for RNA extraction. The dose of anti-IFNT antibody (1:300) was selected on the basis of preliminary experiments, where different dilutions of anti-IFNT antibody (1:300, 500, 1000 and 2000) were used to block effect of IFNT (50 pg/ml) on PBMCs gene expression (data not shown).

### RNA extraction and cDNA synthesis

Total RNA extraction from BOECs and PBMCs was performed using Trizol reagent according to a protocol described previously^47^. Extracted RNA was detected by ultraviolet (UV) spectroscopy (optical density, 260 nm) and concentration was determined by a spectrophotometer (Eppendorf, Munich, Germany) at 260 and 280 nm absorbance values. After measurements, the RNA was stored at −80 °C in RNA storage solution (Ambion, Austin, TX, USA) until cDNA production. The cDNA was synthesized following a protocol described previously^5^ and stored at −30 °C.

### Real-time PCR

The primers of target genes used in the present study are listed in Table 1. Quantitative real-time PCR was carried out by an iCycler iQ (Bio-Rad Laboratories, Tokyo, Japan) using QuantiTect SYBR Green PCR Master Mix (QIAGEN GmbH, Hilden, Germany). The amplification program was run with an initial activation step (15 min at 9 °C), followed by 40 cycles of PCR (15 sec denaturation at 95 °C, 30 sec annealing at 55–58 °C, and 20 sec extension at 72 °C). Melting curve was evaluated at the end of the run to observe the specificity of the amplification. The calculated cycle threshold (Ct) values were normalized using *B-actin* as the internal standard. Fold changes in relative gene expression were determined using the Delta-Delta comparative threshold method^48^.

**Table 1:** List of the primers used in real-time PCR.

Gene		Sequence of nucleotide (5′→3′)	Accession no.	Fragment size (bp)
*B-actin*	Forward	TCACCAACTGGGACGACATG	NM_173979.3	51
	Reverse	CGTTGTAGAAGGTGTGGTGCC		
*ISG15*	Forward	TCTGAGGGACTCCATGACGG	NM_174366	51
	Reverse	TTCTGGGCGATGAACTGCTT		
*OAS1*	Forward	TAGGCCTGGAACATCAGGTC	NM_001040606.1	105
	Reverse	TTTGGTCTGGCTGGATTACC		
*MX2*	Forward	CTTCAGAGACGCCTCAGTCG	NM_173941	232
	Reverse	TGAAGCAGCCAGGAATAGT		
*STAT1*	Forward	CTCATTAGTTCTGGCACCAGC	NM_001077900.1	108
	Reverse	CACACGAAGGTGATGAACATG		
*IFNAR1*	Forward	GCGAAGAGTTTCCGCAACAG	NM_174552.2	275
	Reverse	TCCAAGGCAGGTCCAATGAC		
*IFNAR2*	Forward	TCGTATGTTGCGCCTGTTCT	NM_174553.2	231
	Reverse	GTCCGTCGTGTTTACCCACA		
*PTGES*	Forward	AAAATGTACGTGGTGGCCGT	NM_174443.2	51
	Reverse	CTTCTTCCGCAGCCTCACTT		
*NFKB2*	Forward	CCTGCTGAATGCTCTGTCTG	NM_001102101.1	102
	Reverse	TCCTCCTTCACCTCTGTGCT		
*NFKBIA*	Forward	AAGTGGTCCGCCAAGTGAAG	NM_001045868.1	105
	Reverse	CGATTTCTGGCTGGTTAGTGATC		
*TNFA*	Forward	CAAAAGCATGATCCGGGATG	NM_173966.3	51
	Reverse	TTCTCGGAGAGCACCTCCTC		
*IL1B*	Forward	AATCGAAGAAAGGCCCGTCT	NM_174093.1	51
	Reverse	ATATCCTGGCCACCTCGAAA		
*TGFB1*	Forward	CTGCTGAGGCTCAAGTTAAAAGTG	NM_001166068.1	90
	Reverse	CAGCCGGTTGCTGAGGTAG		
*IL10*	Forward	GAGATGCGAGCACCCTGTCT	NM_174088.1	51
	Reverse	GGCTGGTTGGCAAGTGGATA		
*IL17*	Forward	CACAGCATGTGAGGGTCAAC	NM_001008412	83
	Reverse	GGTGGAGCGCTTGTGATAAT		

### ELISA for determination of PGE2 and IFNT concentration in media

Specific ELISA kits were used for determination of PGE2 (R & D Systems, Minneapolis, MN, USA) and IFNT (Clould-Clone Corpo., Texas, USA) concentrations in the embryo-BOEC co-culture medium, following the manufacturers’ instructions. An ELISA microplate reader (Labsystem Multiskan MS 352, Labsystems, Finland) was used to detect the optical density (OD) value at 450 nm wavelength. The standard curves were prepared in the range of 20–2500 pg/ml and 7.8–500 pg/ml for PGE2 and IFNT, respectively.

### Statistical analysis

SPSS software version 14.0 (SPSS Inc., Chicago, USA) was used for data analysis. Student’s t-test was applied to compare the mean differences between two groups, and one-way ANOVA followed by Tukey’s test was used to compare treatment effects for more than two groups. All values are presented as mean ± standard error of the mean (SEM). Data were considered to be statistically significant at *P* < 0.05.

## Electronic supplementary material

Supplementary figures
