# Supplementary material for: Oviduct epithelium induces interferon-tau in bovine Day-4 embryos, which generates an anti-inflammatory response in immune cells
Source: Sci Rep. 2018 May 18;8:7850. doi: 10.1038/s41598-018-26224-8 (PMC5959944; doi:10.1038/s41598-018-26224-8)
Supplement: Supplementary file 1 — Supplementary figures [file 41598_2018_26224_MOESM1_ESM.pdf]

Oviduct epithelium induces interferon-tau in bovine Day-4 embryos, which generates an anti-inflammatory response in immune cells

Anup K. Talukder[1,2], Mohammad B. Rashid[1,3], Mohamed S. Yousef[1,4], Kazuya Kusama[5], Takashi Shimizu[1], Masayuki Shimada[6], Susan S. Suarez[7], Kazuhiko Imakawa[5] & Akio Miyamoto[1,*]

[1]Graduate School of Animal and Food Hygiene, Obihiro University of Agriculture and Veterinary Medicine, Obihiro 080-8555, Japan

[2]Department of Gynecology, Obstetrics and Reproductive Health, Faculty of Veterinary Medicine and Animal Science, Bangabandhu Sheikh Mujibur Rahman Agricultural University, Gazipur 1706, Bangladesh

[3]Department of Physiology and Pharmacology, Faculty of Veterinary and Animal Science, Hajee Mohammad Danesh Science and Technology University, Dinajpur 5200, Bangladesh

[4]Department of Theriogenology, Faculty of Veterinary Medicine, Assiut University, Assiut, Egypt

[5]Animal Resource Science Center, Graduate School of Agricultural and Life Sciences, The University of Tokyo, Ibaraki 319-0206, Japan

[6]Graduate School of Biosphere Science, Hiroshima University, Higashi-Hiroshima 739-8528, Japan

[7]Department of Biomedical Sciences, Cornell University, Ithaca, NY, 14853, USA

*Corresponding author: akiomiya@obihiro.ac.jp

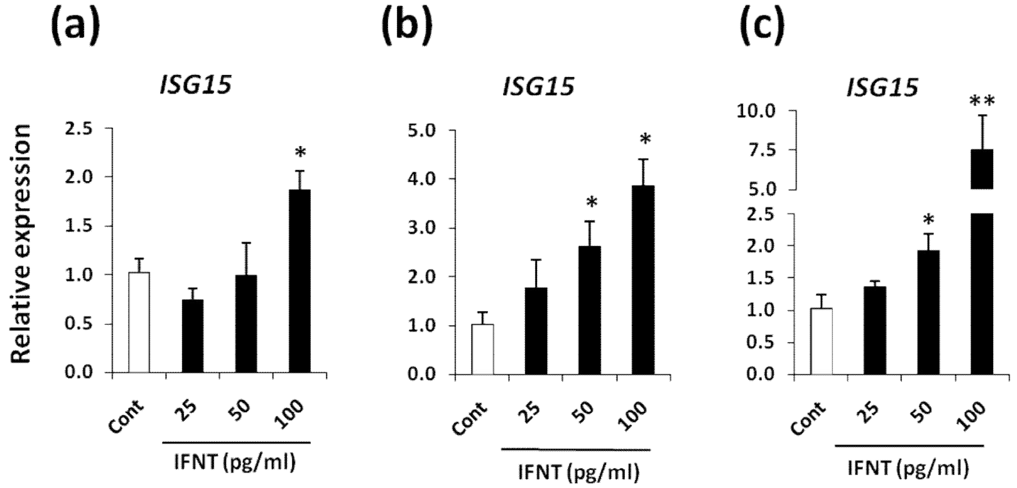

Supplementary Figure 1. (a) Relative mRNA expression of *ISG15* in BOECs exposed to different concentrations of IFNT (25-100 pg/ml) for 24 h. Three to four oviducts from three to four different cows were used for BOECs culture in each experiment. **(b)** Relative mRNA expression of *ISG15* in PBMCs exposed to different concentrations of IFNT (25-100 pg/ml) for 24 h. **(c)** Relative mRNA expression of *ISG15* in PBMCs exposed to IFNT (25-100 pg/ml)-treated BOEC media for 24 h. Data are presented as mean ± SEM of three independent experiments performed in triplicate. * $P<0.05$, ** $P<0.01$, when compared to the control (cont).

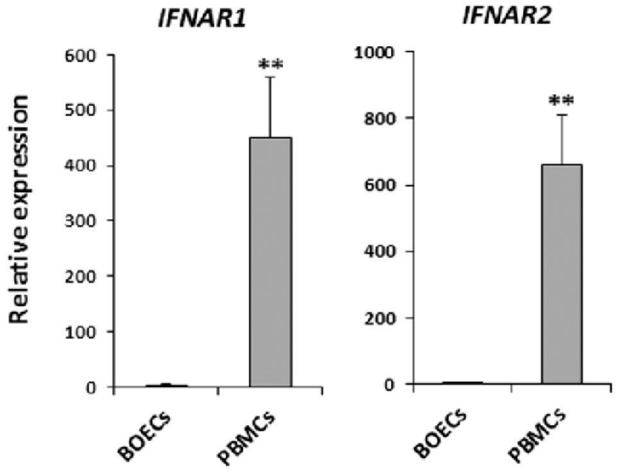

Supplementary Figure 2. Relative mRNA expression of *IFNAR1* and *IFNAR2* in BOECs and PBMCs. Three to four oviducts from three to four different cows were used for BOECs culture in each experiment. Data are presented as mean ± SEM of six independent experiments performed in duplicate. Asterisks denote significant difference: ** *P*<0.01, when compared to the BOECs.

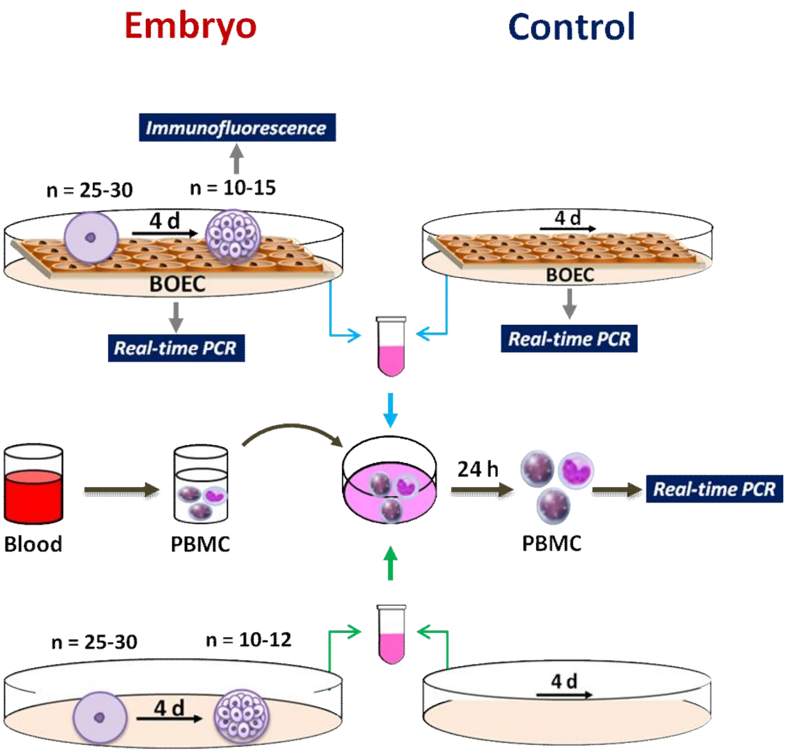

Supplementary Figure 3. Schematic representation of main experimental model. A BOEC monolayer was co-cultured with and without zygotes (control) for 4 days. PBMCs were cultured in embryo-BOEC co-culture medium or BOEC culture medium (control) for 24 h. Next, zygotes were cultured alone without BOECs for 4 days. PBMCs were cultured in medium from embryo culture medium or medium that had been incubated without embryos (control) for 24 h. Embryos were immunolabelled for IFNT. BOECs and PBMCs were analyzed for gene expression by real-time PCR.

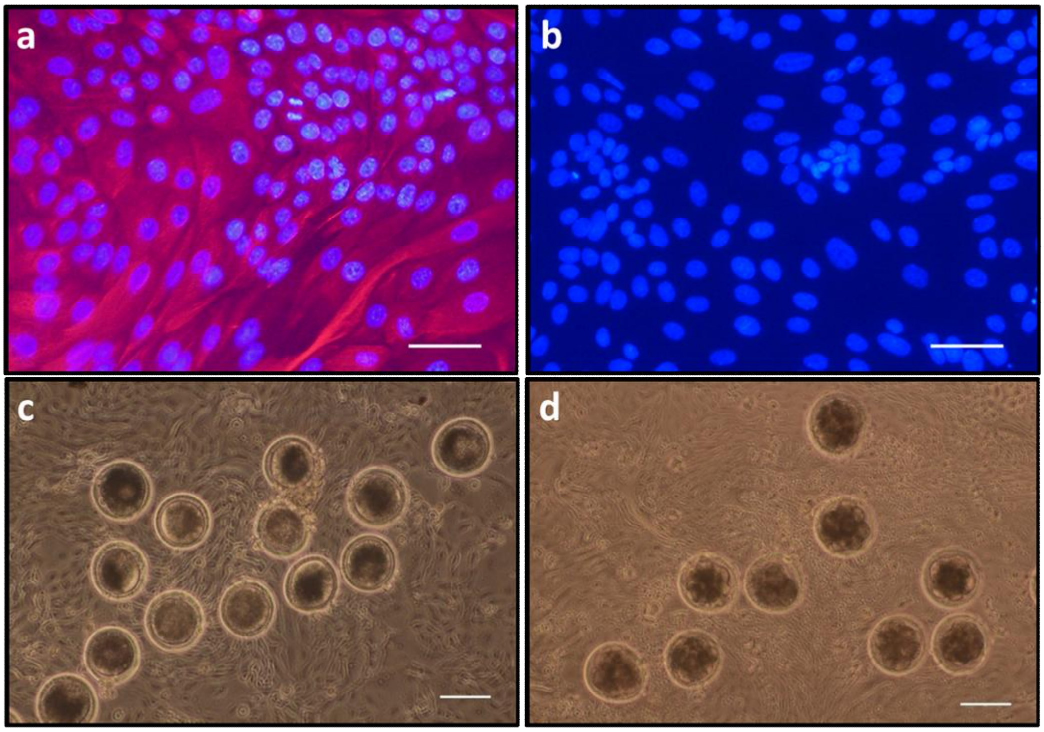

Supplementary Figure 4. (a) Immunofluorescence image of cultured BOECs with anti-cytokeratin antibody. Goat anti-rabbit IgG labelled with Alexa Flour 546 (red) was the secondary antibody; DAPI (blue) was used to visualize nuclei. **(b)** Immunofluorescence image of cultured BOECs without anti-cytokeratin antibody as negative control. PBS-T was used instead of primary antibody. **(c)** Zygotes on the BOEC monolayer at the start of a 4 day co-culture. **(d)** Early morulae on the BOEC monolayer at the end of 4 days of co-culture. Scale bar = 100 µm for a, b and 200 µm for c, d.

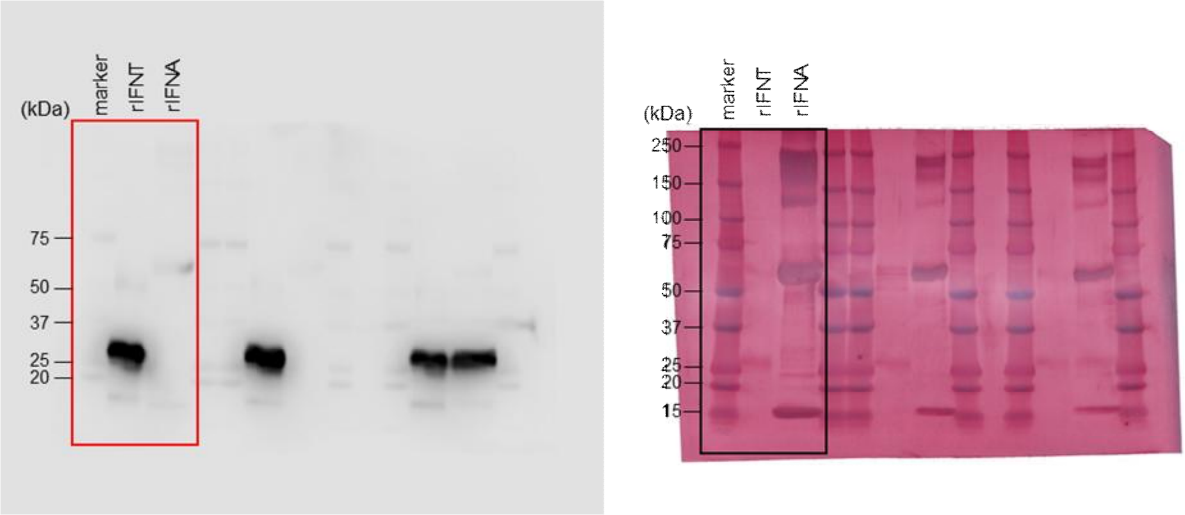

Supplementary Figure 5. Full blots for Figure 1(b). Left: recombinant IFNT, but not recombinant IFNA was detected using anti-IFNT antibody. Right: the PVDF membrane was stained with Colloidal Gold Total Protein Stain solution to confirm total protein loading. It showed bands at the molecular masses of the recombinant proteins.
